# It's time to fear! Interval timing in odor fear conditioning in rats

**DOI:** 10.3389/fnbeh.2013.00128

**Published:** 2013-09-27

**Authors:** Kiseko Shionoya, Chloé Hegoburu, Bruce L. Brown, Regina M. Sullivan, Valérie Doyère, Anne-Marie Mouly

**Affiliations:** ^1^Centre de Recherche en Neurosciences de Lyon, INSERM U1028, CNRS UMR5292, University Lyon1Lyon, France; ^2^Department of Psychology, Queens College and the Graduate CenterNew York, NY, USA; ^3^Child and Adolescent Psychiatry, Emotional Brain Institute, Nathan Kline Institute, New York University School of MedicineOrangeburg, NY, USA; ^4^Centre de Neurosciences Paris-Sud, University Paris-Sud, UMR 8195Orsay, France; ^5^Centre National de la Recherche ScientifiqueOrsay, France

**Keywords:** odor fear conditioning, interval timing, amygdala, dopamine, respiration, ultrasonic vocalization, freezing

## Abstract

Time perception is crucial to goal attainment in humans and other animals, and interval timing also guides fundamental animal behaviors. Accumulating evidence has made it clear that in associative learning, temporal relations between events are encoded, and a few studies suggest this temporal learning occurs very rapidly. Most of these studies, however, have used methodologies that do not permit investigating the emergence of this temporal learning. In the present study we monitored respiration, ultrasonic vocalization (USV) and freezing behavior in rats in order to perform fine-grain analysis of fear responses during odor fear conditioning. In this paradigm an initially neutral odor (the conditioned stimulus, CS) predicted the arrival of an aversive unconditioned stimulus (US, footshock) at a fixed 20-s time interval. We first investigated the development of a temporal pattern of responding related to CS-US interval duration. The data showed that during acquisition with odor-shock pairings, a temporal response pattern of respiration rate was observed. Changing the CS-US interval duration from 20-s to 30-s resulted in a shift of the temporal response pattern appropriate to the new duration thus demonstrating that the pattern reflected the learning of the CS-US interval. A temporal pattern was also observed during a retention test 24 h later for both respiration and freezing measures, suggesting that the animals had stored the interval duration in long-term memory. We then investigated the role of intra-amygdalar dopaminergic transmission in interval timing. For this purpose, the D1 dopaminergic receptors antagonist SCH23390 was infused in the basolateral amygdala before conditioning. This resulted in an alteration of timing behavior, as reflected in differential temporal patterns between groups observed in a 24 h retention test off drug. The present data suggest that D1 receptor dopaminergic transmission within the amygdala is involved in temporal processing.

## Introduction

Time perception is crucial to survival and goal reaching in humans and other animals and interval timing which refers to the ability to time intervals between arbitrary events ranging from seconds to minutes, also guides fundamental animal behaviors. Since Pavlov and Anrep (1927), accumulating experimental evidence has made it clear that in associative learning temporal relations between events are encoded. Balsam and Gallistel (2009), Balsam et al. (2010) proposed that these temporal relations are constantly and automatically encoded and are the foundation of associative learning. They further suggested that this temporal learning occurs very rapidly and prior to the appearance of the anticipatory response Balsam et al. (2010). Yet few studies have addressed this question. Indeed most studies investigating interval timing in animals use peak interval procedures (Bitterman, 1964) or temporal discrimination tasks (Stubbs, 1968) which both necessitate numerous conditioning sessions, thus precluding the observation of timing behavior at the beginning of learning.

Pavlovian fear conditioning is acquired in very few trials. In this paradigm an initially neutral stimulus (the conditioned stimulus, CS) signals the arrival of an aversive unconditioned stimulus (generally a mild foot-shock, US) at a fixed time interval. After a few trials, exposure to the CS alone elicits conditioned responses (e.g., freezing response in rats). A few studies in the literature suggest that the temporal relationship between the CS and the US is learned rapidly, even after a single CS-US pairing (Davis et al., 1989; Arcediano et al., 2003; Drew et al., 2005; Diaz-Mataix et al., 2013b). Most of these studies, however, have used methodologies that do not permit online analysis of the acquisition of the temporal learning over the course of conditioning trials. For example in rodents, fear conditioning studies usually quantify freezing behavior to assess the fear response. While freezing is a robust and easily quantifiable response in rats, it lacks temporal sensitivity and plasticity. Indeed, once induced in response to a foot-shock, freezing often persists throughout the session thus precluding the observation of subtle transient variations in animal's fear levels. There are several examples in the literature highlighting the fact that different behavioral responses may or may not be adequate for measuring learning, as behavioral expression may not always reflect what has been learned (e.g., Brown et al., 1997; Ohyama and Mauk, 2001). In the present study we sought to analyze in parallel different behaviors from the rat's repertoire in order to see whether some of them may show the early learning of the CS-US interval online during Pavlovian fear conditioning.

In a recent study we validated an experimental arrangement designed to record three parameters in parallel in behaving animals: freezing, respiration and ultrasonic vocalizations (USV) (Hegoburu et al., 2011). Respiration rate is known to be modulated by different factors including emotion (Stevenson and Ripley, 1952; Boiten, 1998; Homma and Masaoka, 2008), anticipation of reward (Clarke and Trowill, 1971; Waranch and Terman, 1975), and sampling of odorants (Macrides et al., 1982; Youngentob et al., 1987; Kepecs et al., 2007; Wesson et al., 2008). In addition, changes in respiratory rate can also occur as a conditioned response when motivationally neutral stimuli are contiguously paired with reinforcing stimuli (Freeman et al., 1983; Monod et al., 1989; Nsegbe et al., 1997). USV can also provide information in the context of emotional memory. In rats, USV in the 22 kHz range are observed in dangerous and aversive situations like predator encounter (Blanchard et al., 1991) or painful stimuli (Borta et al., 2006), and can be modulated in anticipation of a negative outcome (Wohr et al., 2005; Portfors, 2007). Interestingly, 22 kHz USV are not restricted to aversive situations as they have also been described in socio-sexual behaviors in male rats (Barfield and Geyer, 1972; McIntosh et al., 1979). Our own data showed that when recorded in parallel, respiration and USV provide complementary information about behavior (Hegoburu et al., 2011).

In the present study we monitored respiration and USV together with freezing behavior in order to perform fine-grain analysis of the rat's fear response during odor fear conditioning. The first aim of our study was to investigate the development of a temporal pattern of responding related to CS-US interval duration, indicating an encoding of time durations in this paradigm. The second aim of the present work was to investigate the role of intra-amygdalar dopaminergic transmission in interval timing in odor fear conditioning. Indeed, learning of Pavlovian associations may depend on prediction error, i.e., detection of unexpected outcome (Rescorla and Wagner, 1972), a process in which time could play a critical role (Diaz-Mataix et al., 2013a). In this context, dopaminergic transmission, as a major player in detection of prediction error (Schultz, 2013), may play a significant role. In addition to its potential role in error detection, dopamine (DA) plays an important role in fear and anxiety through its action in the amygdala (for a recent review see de la Mora et al., 2010) and this has been shown to be true for odor fear conditioning as well (Rosenkranz and Grace, 2002). Furthermore, research on the neural basis of timing has suggested that DA is involved in time perception (Maricq et al., 1981; Meck, 1996; Buhusi and Meck, 2002; Drew et al., 2003). Interestingly, recent studies have proposed that the amygdala could be involved in timing the CS-US interval. For example, in an experiment carried out on behaving cats, Pare and Collins (2000) showed that the responsiveness of amygdala neurons to the tones predicting the footshock increased during the trial, whereas responses to unrelated stimuli remained stable. In another study using auditory fear conditioning in rats, Diaz-Mataix et al. (2013b) showed that changing the interval duration between the CS and the US (from 30 to 10 s) during memory reactivation was sufficient to trigger synaptic plasticity and reconsolidation of the fear memory in the lateral nucleus of the amygdala. In the present study, we therefore investigated the effect of the blockade of DA transmission in the basolateral amygdala on CS-US interval timing in odor fear conditioning. Since the D1 receptor is the main type of dopamine receptors in the basolateral amygdala (Dawson et al., 1986; Scibilia et al., 1992), we chose to infuse the D1 receptor antagonist SCH23390 in this structure.

## Methods

### Animals

Data were obtained from thirty-eight male Long Evans rats (Janvier, France), 14 in Experiment 1 and 24 in Experiment 2, weighing 250–300 g at the start of the experimentation. They were housed individually at 23°C and maintained under a 12 h light–dark cycle (lights on from 7:00 am to 7:00 pm). Food and water were available *ad libitum*. All experiments and surgical procedures were conducted in strict accordance with the European Community Council Directive of November 24, 1986 (86/609/EEC) and the French National Committee (87/848) for care and use of laboratory animals. The experiments were carried out under the approval of Direction of Veterinary Service (#69000692), and care was taken at all stages to minimize stress and discomfort to the animals.

### Experimental apparatus

The apparatus has been described in a previous study (Hegoburu et al., 2011). It consisted of a whole body customized plethysmograph (diameter 20 cm, height 30 cm, Emka Technologies, France) placed in a sound-attenuating cage (L 60 cm, W 60 cm, H 70 cm). The plethysmograph was used to measure respiratory parameters in behaving animals. The ceiling of the plethysmograph was equipped with a tower which allowed the introduction of three Tygon tubing (diameter 3 mm) connected to a programmable custom olfactometer to deliver air and odorants. Deodorized air flowed constantly through the cage (2 L/min). When programmed, an odor (McCormick Pure Peppermint; 2 L/min; 1:10 peppermint vapor to air) was introduced smoothly in the air stream through the switching of a solenoid valve (Fluid automation systems, CH-1290 Versoix, Switzerland) thus minimizing its effect on change in pressure. The solenoid valve was inserted in a sound-attenuating box in order to prevent perception by the animals of any sound from the olfactometer which could provide an additional timing cue. The bottom of the animal chamber had a port connected to a ventilation pump which could draw air out of the plethysmograph (at a rate of up to 2 L/min) thus maintaining a constant airflow that did not interact with the animal's breathing pattern. A condenser ultrasound microphone (Avisoft-Bioacoustics CM16/CMPA, Berlin, Germany) was inserted in the tower on the top of the plethysmograph to monitor USV emitted by the rats. The bottom of the animal chamber was equipped with a shock floor connected to a programmable Coulbourn shocker (Bilaney Consultants GmbH, Düsseldorf, Germany). Animal's behavior was monitored with four video cameras (B/W CMOS PINHOLE camera, Velleman, Belgium) placed at each corner of the sound-attenuating cage.

### Odor fear conditioning paradigm

During the four days preceding conditioning, the animals were handled and familiarized with the conditioning cage for 20 min each day.

**Figure 1:**
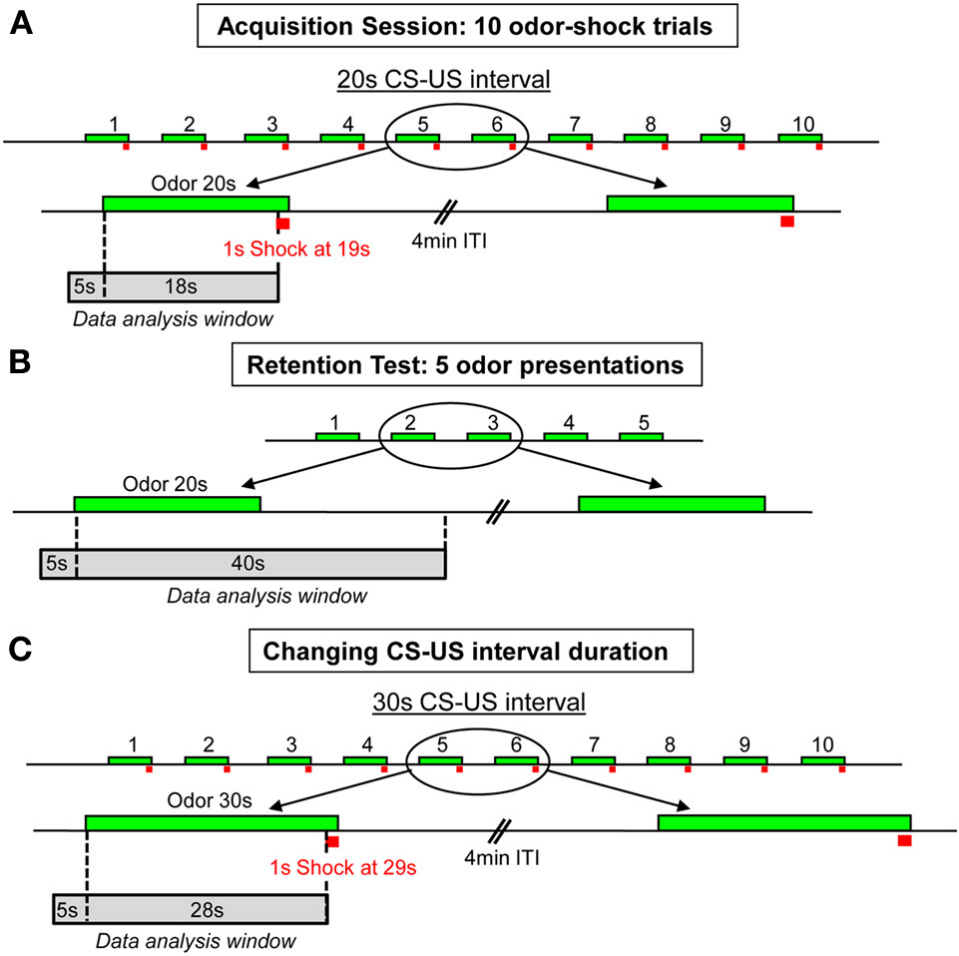
**Schema of the experimental protocol used for the odor fear conditioning paradigm. (A)** Acquisition session: 10 odor-shock pairings were delivered with a 4 min intertrial interval (ITI). The CS-US interval duration was set at 20 s. **(B)** Retention test: 24 h after training, five CS odor presentations were carried out with a 4 min intertrial interval (ITI). **(C)** Change in CS-US interval duration: in the second part of the experiment, ten odor-shock pairings were applied with a new 30 s CS-US interval duration. The green boxes symbolize the CS odor, the red squares represent the footshock, and the gray boxes indicate the time window during which the analysis of the recorded parameters has been performed.

In Experiment 1, two experimental conditions were used: Odor-shock pairings (Paired group) and Odor-alone presentations (Odor group). In the Paired group (*n* = 8), during the first 4 min of the conditioning session, the animals were allowed free exploration, then the CS odor was introduced into the cage for 20 s, the last second of which overlapped with the delivery of a 0.4 mA foot-shock (Figure 1A). The CS odor did not end abruptly after the odor valve switched-off at 20 s. It remained perceptible (with a progressively decaying concentration) for approximately 20 additional seconds based on the experimenter's olfactory judgment. The animal received ten odor-shock trials, with an intertrial interval of 4 min. After the last pairing, the animal was left in the conditioning cage for 8 min, after which it was returned to its home cage. In the Odor group (*n* = 6), the same procedure was carried out except that the odor was presented alone. The conditioned fear response was assessed during a retention test carried out 24 h after the acquisition session. For the retention test, the rat was placed in the experimental cage and allowed a 4-min odor-free period. The CS odor was then presented five times for 20 s with a 4-min intertrial interval (Figure 1B). One week after the retention test, Paired animals were trained again, using a new CS-US interval duration (Figure 1C). The animals received ten odor-shock trials, with the odor delivered for 30 s and the shock arriving during the last second. During the different steps of the experiment, the animal's behavior, respiration, and USV production were continuously monitored for offline analysis.

In Experiment 2, the effect of the injection of the D1 receptor antagonist SCH23390 in the amygdala on the animals' performances in odor fear conditioning was assessed. Two experimental conditions were used with all the animals trained as described for the Paired group in Experiment 1 (Figures 1A,B). In the SCH23390 group (*n* = 11), the animals received an infusion of SCH23390 5 min prior to the acquisition session while in the NaCl group (*n* = 13), the animals received an infusion of NaCl. 24 h later, the two groups were tested for their retention of the learning as described in Experiment 1.

### Surgery and drug administration

In Experiment 2, the animals were anesthetized with ketamine (70 mg/kg) and xylazine (6 mg/kg) administrated by intraperitoneal injection, and placed in a stereotaxic frame (Narishige, Japan). Before head skin incision, bupivacaine (1% solution; Sigma-Aldrich, Saint-Quentin Fallavier, France) was administered subcutaneously for local anesthesia. During the surgery, the animal's rectal temperature was maintained at 37–38°C with a servo-controlled heat blanket (Harvard Apparatus, Phymep, France). After surgery and at all stages of the experiment care was taken to minimize animal's suffering. The rats were implanted bilaterally with stainless steel guide cannulae (23G × 12 mm, Phymep, France) in the basolateral amygdala (stereotaxic coordinates from Paxinos and Watson, 1998: 2.8 mm posterior to bregma, 4.9 mm from midline and 6.0 mm ventral from dura). The tips of the cannulae were aimed 1.5 mm above the intended area. The cannulae were fixed to the skull with dental acrylic cement and anchored with a surgical screw placed in the skull. Stylets were inserted into the guide cannulae to prevent clogging. The animals were allowed 1 week of post-surgical recovery. All animals were handled individually for 5 min each day during the last 3 days before infusion in order to familiarize them with being manipulated. During infusion, the rats were gently held on the experimenter's arm, stylets were removed, and injection needles (30G) were inserted, extending 1.5 mm from the tip of the guide cannula. The injection needles were connected via polyethylene tubing to two 10-mL Hamilton microsyringes driven by an automated microinfusion pump (Harvard Apparatus, France). The D1 receptor antagonist SCH23390 (3 μg/0.5 μl, Sigma-Aldrich France) was dissolved in sterile saline (NaCl), which was used as control. A total volume of 0.5 μL per hemisphere was delivered over 1 min. After the injection, the needles were left in position for an additional minute to enable diffusion of the solution into the tissue. At the end of the experiment, the animals were sacrificed with a lethal dose of pentobarbital, and the placement of the injection canulae tips was verified.

### Data acquisition and pre-processing

#### Respiration recording

The respiratory signal collected from the plethysmograph was amplified and sent to an acquisition card (MC-1608FS, Measurement Computing, USA; Sampling rate = 1000 Hz) for storage and offline analysis. The detection of the respiratory cycles was achieved using an algorithm described in a previous study (Roux et al., 2006). This algorithm performs two main operations: signal smoothing for noise reduction, and detection of zero-crossing points in order to define accurately the inspiration and expiration phase starting points. Momentary respiratory frequency was determined as the inverse of the respiratory cycle (inspiration plus expiration) duration.

#### USV recording

The ultrasound microphone was connected to a recording interface (UltraSoundGate 116 Hb, Avisoft-Bioacoustics) with the following settings: sampling rate = 214285 Hz; format = 16 bit (Wohr et al., 2005). Recordings were transferred to Avisoft SASLab Pro (version 4.2, Avisoft Bioacoustics, Berlin, Germany) and a Fast Fourier Transform (FFT) was conducted. Spectrograms were generated with an FFT-length of 512 points and a time window overlap of 87.5% (100% Frame, FlatTop window). These parameters produced a spectrogram at a frequency resolution of 419 Hz and a time resolution of 0.29 ms. The acoustic signal detection was provided by an automatic whistle tracking algorithm with a threshold of −20 dB, a minimum duration of 0.01 s and a hold time of 0.02 s. However, the accuracy of detection was verified trial by trial by an experienced user. The main parameters used in the present study were the duration as well as the start and end time of USV calls. No band pass filter has been applied during USV recording. Ninety-three percent of the recorded USV calls were in the 21–27 kHz range (with a maximum of calls around 24 KHz) which is in accordance with previous data from the literature (Wohr et al., 2005). USVs in the 45–60 kHz range represented less than 1% of the total.

Because USV emission is not systematically observed in response to an aversive stimulus (Borta et al., 2006), only animals producing USV in the paired groups were included in this analysis. This number is specified for the Paired group in each analysis. For the Odor group, the number of animals was always 6.

#### Behavior coding

The video signal collected through the four video cameras was acquired with homemade acquisition software using the Matrox Imaging Library and a Matrox acquisition card (Morphis QxT 16VD/M4, Matrox video, UK). Offline, the video recordings were replayed and the animal's freezing behavior was encoded by an experimenter blind to the condition, using an ethogram keyboard.

### Data synchronization and analysis

The different data (respiration, USV, behavior) were then entered in a database (Garcia and Fourcaud-Trocme, 2009). The first step of data analysis was data synchronization. This was achieved via a TTL synchronization signal generated at the beginning of each experimental session. Secondary TTL signals were also generated for important events in the session: odor arrival, shock delivery. Once synchronized, the data were analyzed using scripts in Python. The simultaneous time course of respiration, USV, and behavior was assessed in parallel throughout the experimental session. For this, instant respiratory frequency, freezing and USV rates were averaged on a second by second basis. The resulting individual curves were then averaged among animals of the same experimental group.

### Statistical analysis

Two types of analyses were performed. A first analysis was carried out to assess the global effects of conditioning on the average value of the different parameters. For this, for each parameter individually, the average value of the 5 s preceding odor delivery (Pre-CS period) was compared with the average value of the 18 s (or 28 s) between odor onset and shock delivery (CS period) for the acquisition session, or the 40 s following odor onset (duration of the odor perception) for the retention session (see on Figure 1, data analysis windows). We used a Two Way ANOVA with the group as an independent factor and the period (Pre-CS *vs.* CS) as a repeated measures factor. *Post-hoc* Tukey comparisons were then carried out when allowed by the ANOVA results. A second analysis was performed to assess the effects of conditioning on the temporal dynamics of the recorded parameters in presence of the CS odor. For this analysis, the 1-s time course of each parameter from the odor onset to 18 s after was compared using a Two-Way ANOVA with the group as an independent factor and the time as a repeated measures factor. For this analysis, the 1-s time course of each parameter during the CS-US interval (1–18 s or 1–28 s) for acquisition, and in response to the CS odor (1–40 s) for retention, was compared using a Two-Way ANOVA with the group as an independent factor and the time as a repeated measures factor. *Post-hoc* pairwise comparisons were then carried out when allowed by the ANOVA results. For all the statistical comparisons performed, the significance level was set at 0.05.

## Results

### Experiment 1: behavioral assessment of interval timing in odor fear conditioning

In this experiment, we analyzed the effects of conditioning on (1) the average global value of the recorded parameters when comparing pre-Cs vs. CS period and (2) their fine-grain temporal pattern during the CS odor. These effects were assessed at different stages of the procedure: acquisition, 24 h retention, and following a change in CS-US interval duration.

#### Acquisition

Three parts of the acquisition session were considered: Trial 1, Trials 2–5, and Trials 6–10. In the two latter cases, the average curves of the individual trials were pooled together.

**Figure 2:**
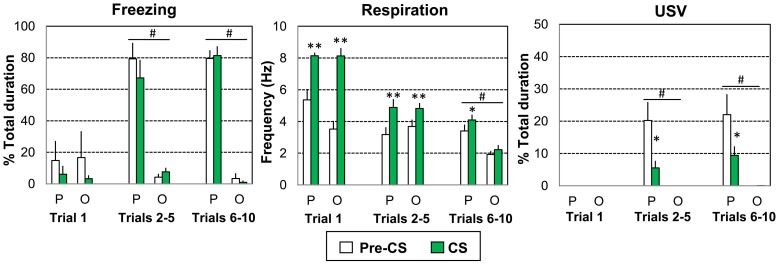
**Global effect of odor-shock conditioning on freezing, respiration, and ultrasonic vocalization (USV)**. The Paired group (P, *n* = 8) received 10 odor (20 s)-shock (1 s) pairings while the Odor group (O, *n* = 6) received 10 odor (20 s) presentations. Three parts of the acquisition session were considered: Trial 1, Trials 2–5, and Trials 6–10. The data for freezing duration, respiration frequency, and USV duration were averaged for the 5 s preceding odor onset (Pre-CS period) and compared to the average of the 18 s between odor onset and shock delivery (CS period). ^#^significant difference between the two experimental groups (*p* = 0.001); ^*^significant difference with Pre-CS period (^*^p < 0.05; ^**^p < 0.005).

***Global effect of conditioning.*** To assess the global level of conditioning, we first analyzed how learning affected the mean values of the recorded parameters over the course of the training trials. For this purpose, the data for each parameter were averaged for the 5 s preceding odor onset (Pre-CS period) and compared to the average of the 18 s between odor onset and shock delivery (CS period; Figure 2). A Three-Way ANOVA with two repeated measures (Trials and Period) was performed for each dependent variable.

For freezing (Figure 2, left) the ANOVA revealed a significant effect of Group [*F*_(1, 12)_ = 34.858, *p* < 0.001], Trials [*F*_(2, 24)_ = 19.471, *p* < 0.001] and Trials × Group interaction [*F*_(2, 24)_ = 28.228, *p* < 0.001], but no significant effect of period or related interactions. A strong increase in freezing was observed in the Paired group for Trials 2–5 and Trials 6–10 (p < 0.001 compared to the Odor group), during both the Pre-CS and CS periods (*p* < 0.005 compared to the first trial values).

Concerning respiration (Figure 2, middle), the ANOVA revealed a significant effect of Trials [*F*_(2, 24)_ = 53.354, *p* < 0.001], Trials × Group interaction [*F*_(2, 24)_ = 4.162, *p* = 0.028], Period [*F*_(1, 12)_ = 50.716, *p* < 0.001], Trials × Period interaction [*F*_(2, 24)_ = 45.119, *p* < 0.001], Trials × Period × Group interaction [*F*_(2, 24)_ = 7.349, *p* = 0.003]. Over the course of the session, respiration frequency decreased faster for the Odor group (due to habituation) than for the Paired group. In both groups the odor induced an increase in respiratory frequency (compared to pre-CS levels) for Trial 1 and Trials 2–5 (*p* < 0.005). For Trials 6–10, the odor induced an increase in frequency only in Paired animals (*p* < 0.05).

Concerning USV (Figure 2, right), odor animals emitted no USV throughout the entire session. In contrast, 5 out of 8 animals in the Paired group produced USV calls. The ANOVA showed a significant effect of Group [*F*_(1, 9)_ = 24.93, *p* = 0.001], Trials [*F*_(2, 18)_ = 8.297, *p* = 0.003] and Period [*F*_(1, 9)_ = 18.732, *p* = 0.002], as well as the following interactions: Trials × Group interaction [*F*_(2, 18)_ = 8.293, *p* = 0.003], Period × Group interaction [*F*_(1, 9)_ = 18.745, *p* = 0.002], Trials × Period interaction [*F*_(2, 18)_ = 9.13, *p* = 0.002], Trials × Period × Group interaction [*F*_(2, 18)_ = 9.134, *p* = 0.002]. No USV calls were detected in either group on the first trial. In Paired animals only, USV calls were observed during the Pre-CS period for Trials 2–5 and Trials 6–10. In both cases, USV emission decreased strongly in presence of the CS odor (*p* < 0.005).

In summary, paired animals presented higher levels of freezing than odor animals (Trials 2–5 and Trials 6–10) both before and during the CS odor presentation. They also exhibited a higher respiratory rate (Trials 6–10) and higher levels of USV emission (Trials 2–5 and Trials 6–10). In addition, in paired animals, introduction of the odor cue resulted in an increase in respiratory rate (Trials 6–10) and in a decrease in USV emission (Trials 2–5 and Trials 6–10) compared to pre-CS period while no changes were observed in odor animals at the same trials.

***Temporal dynamics of the recorded parameters during the odor-shock interval.*** We then examined the temporal pattern during the CS for each parameter in 1-s time bins, from the odor onset to shock delivery (i.e., from 1 to 18 s).

**Figure 3:**
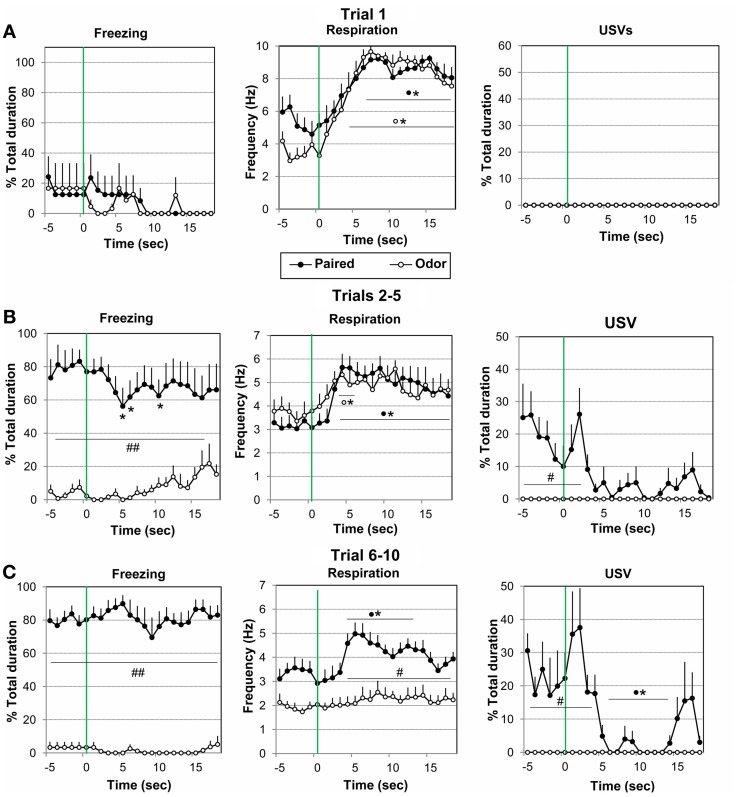
**Effect of odor-shock conditioning on the fine-grain temporal pattern of freezing, respiration, and ultrasonic vocalization (USV) during odor presentation**. The temporal pattern for each parameter is represented with a 1-s bin precision, from 5 s preceding odor onset (green vertical line on each graph) to 18 s after (corresponding to the CS-US interval duration). Paired group: black filled circles (*n* = 8); Odor group: empty circles (*n* = 6). Three parts of the acquisition session were considered: **(A)** Trial 1 (beginning of the session before shock delivery), **(B)** Trials 2–5, and **(C)** Trials 6–10. ^#^Significant difference between the two experimental groups (^##^*p* < 0.001; ^#^*p* < 0.05); ^*^significant difference with pre-odor baseline level (*p* < 0.05 at least). The symbol preceding the asterisk refers to the experimental group.

*Trial 1.* Concerning freezing behavior (Figure 3A, left), the ANOVA revealed no effect of Group, Time, or Group × Time interaction. Freezing behavior was low in both groups.

Concerning respiration (Figure 3A, middle), the ANOVA revealed a significant effect of Time [*F*_(17, 204)_ = 8.66, *p* < 0.001], but no effect of Group or Group × Time interaction. Further within groups comparisons showed that in both groups, arrival of the odor induced a significant increase in respiratory frequency from 4 to 18 s after odor delivery in the Odor Group, and from 6 to 18 s in the Paired Group, as compared to baseline level (*p* < 0.05).

No USV were detected in presence of the odor in either group (Figure 3A, right).

*Trials 2–5*. Concerning freezing behavior (Figure 3B, left), the ANOVA revealed a significant effect of Group [*F*_(1, 12)_ = 19.79, *p* = 0.001], and Group × Time interaction [*F*_(17, 204)_ = 1.96, *p* = 0.015]. In the Paired group freezing rate was significantly higher than in the Odor group before odor delivery due to previous odor-shock pairings (*p* < 0.001), and decreased significantly following odor introduction compared to baseline levels (*p* < 0.05, at 5–6 s and 10 s). Odor animals showed almost no sign of freezing and spent most of the time exploring or remaining immobile.

For respiration (Figure 3B, middle), the ANOVA revealed a significant effect of Time [*F*_(17, 204)_ = 5.22, *p* < 0.001], but no effect of Group or Group × Time interaction. Within group comparisons revealed that Paired animals' respiratory rate increased significantly compared to baseline level, from 3 s after odor onset until shock arrival (*p* < 0.05). Odor animals presented a shorter significant increase in respiratory frequency in response to the odor (*p* < 0.05, from 3 to 5 s after odor delivery), after which respiratory rate returned to pre-odor baseline level.

Concerning USV emission (Figure 3B, right), the ANOVA revealed a significant effect of Group [*F*_(1, 9)_ = 7.99, *p* = 0.020], Time [*F*_(17, 153)_ = 3.72, *p* < 0.001], and Group × Time interaction [*F*_(17, 153)_ = 3.72, *p* < 0.001]. Between groups comparisons showed that there was a significantly higher (*p* < 0.05) USV production rate in Paired animals before odor arrival (from −5 to 3 s). In presence of the odor, USV rate decreased in Paired animals and was not significantly different from Odor animals.

*Trials 6-10*. Concerning freezing behavior (Figure 3C, left), the ANOVA revealed a significant effect of Group [*F*_(1, 12)_ = 141.85, *p* < 0.001], but no effect of Time or Group × Time interaction. *Post-hoc* comparisons showed that in the Paired group freezing rate was significantly higher than in the Odor group before odor delivery, and did not vary significantly in presence of the odor, although a tendency to decrease was observed around 9 s. Odor animals showed almost no sign of freezing and spent most of the time sleeping during trials 6–10.

For respiration (Figure 3C, middle), the ANOVA revealed a significant effect of Group [*F*_(1, 12)_ = 15.95, *p* = 0.002], Time [*F*_(17, 204)_ = 5.14, *p* < 0.001], and Group × Time interaction [*F*_(17, 204)_ = 2.80, *p* < 0.001]. *Post-hoc* comparisons showed that the respiratory rate was globally significantly lower in Odor animals than in Paired animals. In addition, while Odor animals showed almost no reaction upon odor arrival, Paired animals' respiration rate exhibited a temporal pattern with an increase reaching significant levels from 4 s after odor onset until shock arrival (peak frequency at 5–6 s), after which it returned to pre-odor baseline level showing a U-shaped evolution just before shock arrival.

Concerning USV emission (Figure 3C, right), the ANOVA revealed a significant effect of Group [*F*_(1, 9)_ = 13.93, *p* < 0.001], Time [*F*_(17, 153)_ = 5.76, *p* < 0.001], and Group × Time interaction [*F*_(17, 153)_ = 5.76, *p* < 0.001]. In contrast to Odor animals, Paired animals produced USV calls during the intertrial period. *Post-hoc* comparisons showed that there was a significant decrease (*p* < 0.05) in USV production rate upon odor delivery (from 6 to 14 s) followed by a transient inverted U-shaped re-increase toward the pre-CS level before shock arrival (from 15 to 17 s).

In summary, in Paired animals a temporal response pattern was observed within the odor-shock interval for respiration and USV during trials 6–10. Indeed introduction of the learned odor first induced an increase in respiratory rate and a decrease in USV. Then a decrease in respiratory frequency and an increase in USV emission were observed just prior to shock delivery. In contrast freezing rate did not exhibit a systematic temporal pattern throughout odor presentation.

#### Retention test

The animals were tested 24 h after the conditioning session for their retention of the learning. Five presentations of the learned odor were carried out and the five resulting curves were averaged for each parameter.

***Global effect of conditioning.*** For each parameter the data were averaged for the 5 s preceding odor onset (Pre-CS period) and compared to the average of the 40 s after odor onset (CS period, which represents the approximate duration of the odorant stimulus, as judged by the experimenter). A Two Way ANOVA with one repeated measures factor (Period) was performed.

**Figure 4:**
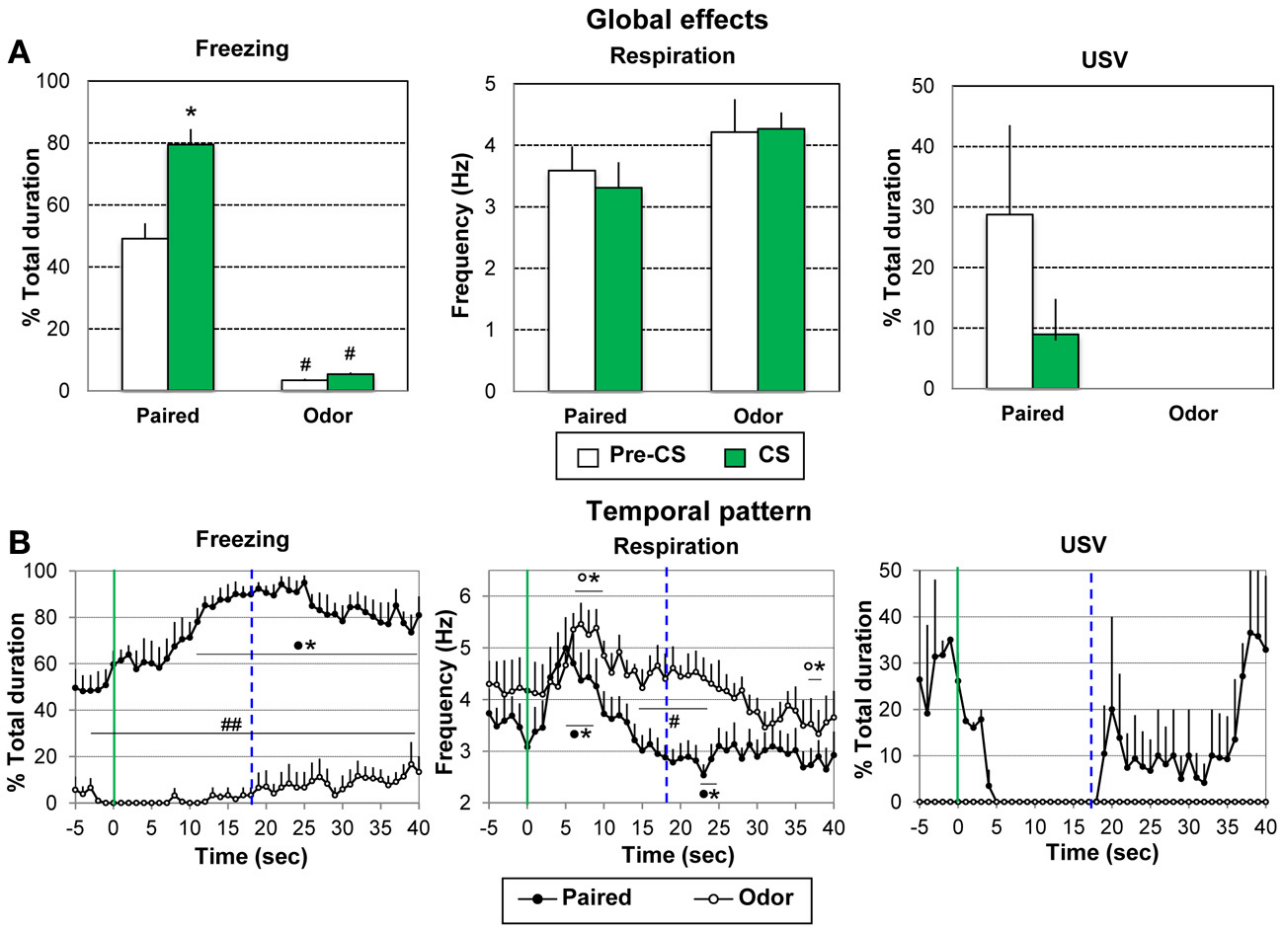
**Effect of odor-shock conditioning on freezing, respiration, and ultrasonic vocalization (USV) assessed during the retention session**. During the retention session, the Paired group (*n* = 8) and the Odor group (*n* = 6) received 5 presentations of the CS odor. The data were averaged over the 5 presentations. **(A)** Global effect: the data for freezing, respiration, and USV were averaged for the 5 s preceding odor onset (Pre-CS period) and compared to the average of the 40 s after odor onset (CS period, which represents the approximate duration of the odorant stimulus, as judged from the experimenter nose). #: significant difference with the equivalent period in the paired group (*p* < 0.001); ^*^significant difference with Pre-CS period (*p* = 0.001). **(B)** Temporal pattern: the temporal pattern for each parameter is represented with a 1-s bin precision, from 5 s preceding odor onset (green vertical line on each graph) to 40 s after. The blue vertical dashed line represents the time of shock arrival in the acquisition session. Paired group: black filled circles (*n* = 8); Odor group: empty circles (*n* = 6). ^#^significant difference between the two experimental groups (^##^*p* < 0.001; ^#^*p* < 0.05); ^*^significant difference with pre-odor baseline level (*p* < 0.05 at least). The symbol preceding the asterisk refers to the experimental group.

For freezing (Figure 4A, left), the ANOVA showed a significant effect of Group [*F*_(1, 12)_ = 104.925, *p* < 0.001], Period [*F*_(1, 12)_ = 26.106, *p* < 0.001] and Period × Group interaction [*F*_(1, 12)_ = 20.099, *p* = 0.001]. In the Paired group, the level of freezing was higher than in the odor group during the Pre-CS and CS periods (*p* < 0.001). In addition the CS induced a further increase in freezing in Paired animals (*p* = 0.001).

Concerning respiration (Figure 4A, middle), no significant effect of Group, Period or Period × Group interaction was observed.

For USV (Figure 4A, right), animals in the Odor group produced no USV calls throughout the entire retention session. In the Paired group, only 2 out of 8 animals produced USV calls. Although no statistical test can be achieved on Paired animals due to the too small number of animals, there was a tendency for the introduction of the odor cue to induce a decrease in USV emission.

When the foregoing analyses were restricted to the 18-s CS-US interval, the conclusions concerning significant effects were unchanged.

***Temporal dynamics of the recorded parameters during odor presentation.*** We then examined the temporal pattern for each parameter with a 1-s bin precision, from the odor onset to 40 s after.

Concerning freezing behavior (Figure 4B, left), the ANOVA revealed a significant effect of Group [*F*_(1, 12)_ = 152.82, *p* < 0.001], Time [*F*_(39, 468)_ = 4.24, *p* < 0.001], and Group × Time interaction [*F*_(39, 468)_ = 2.26, *p* < 0.001]. Between groups comparisons showed that freezing rate was significantly higher in Paired animals than in Odor animals throughout the entire period. Within-group comparisons revealed that in the Paired group, freezing increased significantly in response to the learned odor, from 9 s after odor onset until the end of the time window, with a maximum value around 22–25 s.

For respiration (Figure 4B, middle), the ANOVA revealed a tendency for Group [*F*_(1, 12)_ = 4.43, *p* = 0.057], a significant effect of Time [*F*_(39, 468)_ = 8.94, *p* < 0.001], and a Group × Time interaction [*F*_(39, 468)_ = 2.14, *p* < 0.001]. Within group comparisons showed that in the Paired group, introduction of the odor cue led to a significant (*p* < 0.05) increase in respiratory rate from 3 to 8 s after odor onset. The respiratory frequency then decreased below baseline level to reach a minimum at 23–24 s after odor onset (*p* < 0.05). In the Odor group, the odor-induced increase in respiratory rate occurred slightly later than in the Paired group (6 to 9 s) after which the respiratory frequency returned to pre-odor baseline levels and lower at 38–40 s. Between groups comparisons showed that the respiratory rate was significantly higher in the Odor group from 14 to 24 s after odor onset (*p* < 0.05).

Concerning USV emission (Figure 4B, right), animals in the Odor group produced no USV throughout the entire retention session. In the Paired group, only 2 out of 8 animals produced USVs during the session. Although no statistical test can be achieved on these data due to the small number of animals, it is interesting to notice that the time course of the USV in these animals is similar to the one observed during the training session, with a suppression of USVs following odor onset followed by a re-increase around the expected time of shock arrival (19–20 s).

When the foregoing analyses were restricted to the 18-s CS-US interval, the conclusions regarding significant effects were unchanged.

In summary, during the retention test in paired animals, introduction of the conditioned odor cue induced an increase of freezing with maximum values around the expected time of shock arrival and an increase in respiratory rate followed by a decrease presenting a minimum value around the expected time of shock arrival.

#### Changing interval duration

In order to assess whether the behavioral changes observed just prior to shock arrival during the acquisition session were related to interval timing, the Paired animals were trained for 10 additional trials using a 30 s Odor-shock interval instead of the 20 s interval previously used. The average curves (last five odor-shock trials) for the two durations are represented in Figure 4, from 5 s before the odor onset until shock arrival. A One Way repeated measure ANOVA was carried out on the 30 s curve data. In order to specifically assess the temporal pattern within the CS, the ANOVA was restricted to the 1–28 s interval (corresponding to the CS-US interval duration).

**Figure 5:**
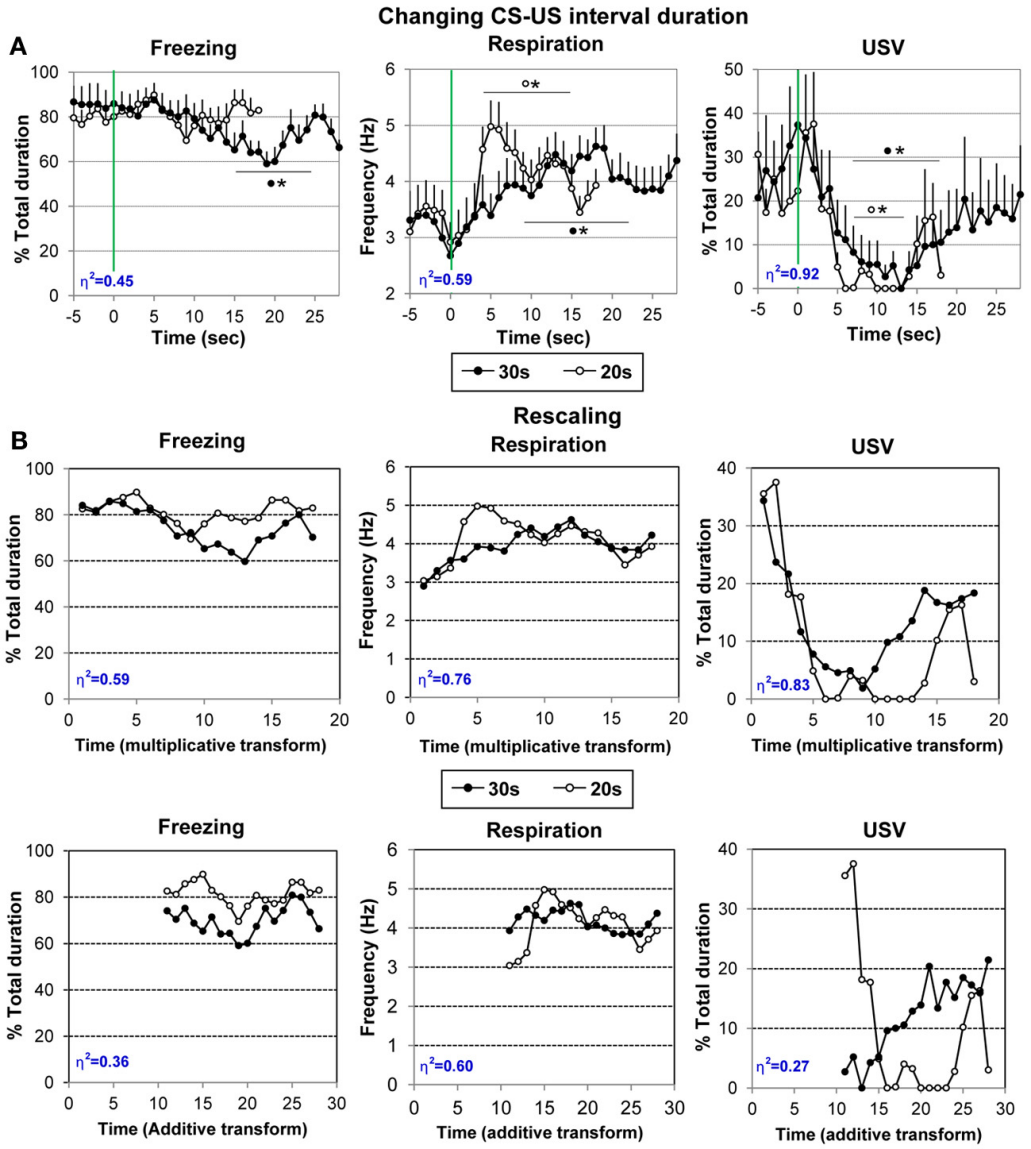
**(A)** Effect on changing CS-US interval duration on the temporal pattern of freezing, respiration and ultrasonic vocalization (USV). The animals of the Paired group were trained for 10 additional trials using a 30 s Odor-shock interval instead of the 20 s interval previously used. The average curves (last five odor-shock trials) for the two CS-US interval durations are represented (30 s: black filled circles; 20 s: empty circles) for each parameter from 5 s before odor onset to the last second prior to shock arrival (18 s or 28 s). ^#^significant difference between the two durations (*p* < 0.05); ^*^significant difference with pre-odor baseline level (*p* < 0.05 at least). The symbol preceding the asterisk refers to the experimental group. **(B)** Data rescaling: the group mean functions were rescaled for both 20- and 30-s conditions, separately for freezing, respiration, and USV. The upper panels present the multiplicative transform in the time axis, while the lower panels present the additive transform in the axis, laterally shifted to represent the same absolute distance from shock in each of the two functions. Superposition between the two curves was indexed by eta-squared (η^2^) indicated in blue in the lower left part of each graph.

Concerning freezing behavior (Figure 5A, left), the ANOVA revealed a significant effect of Time [*F*_(27, 189)_ = 3.01, *p* < 0.001]. Further comparisons showed that freezing decreases significantly from 14 to 23 s after odor onset, to return to pre-odor baseline levels before shock delivery.

For respiration (Figure 5A, middle), the ANOVA revealed a significant effect of Time [*F*_(27, 189)_ = 5.22, *p* < 0.001]. Further comparisons revealed that introduction of the CS odor led to a significant (*p* < 0.05) increase in respiratory rate from 6 to 21 s after odor onset. The respiratory frequency then decreased to pre-odor baseline level showing a U-shaped evolution before shock arrival.

Regarding USV emission (Figure 5A, right), 6 out of 8 animals produced USVs during the session. The ANOVA revealed a significant effect of Time [*F*_(27, 135)_ = 2.42, *p* < 0.05]. Further comparisons showed that odor arrival induced a significant decrease in USV production rate from 7 to 18 s (*p* < 0.05) after which a return to pre-CS baseline level was observed before shock arrival (from 19 to 28 s).

Therefore, changing the odor-shock interval duration resulted in a shift toward the new duration of the anticipatory responses previously observed prior to shock arrival for respiration and USV. In addition, increasing the number of pairings favored the development of an anticipatory response for freezing behavior.

Figure 4A suggests that the temporal patterns of performance under 20-s and 30-s CS-US intervals were similar in shape in relative time, suggesting that the CS-US interval was timed according to the scalar rule (Gibbon, 1997) in both conditions. In order to assess scalar timing quantitatively, the time axis for the 30-s data was multiplicatively rescaled so that the 18 time bins during CS-alone for both groups represented the same proportions of elapsed time from CS onset to shock presentation. The scalar timing rule predicts superior superposition of the functions in relative time, compared to no rescaling or rescaling by an additive shift in axes. Figure 4B shows the rescaled group mean functions for both 20- and 30-s conditions, separately for each behavioral measure. The upper panels present the multiplicative transform of the time axis, while the lower panels present the additive transform in the axis, laterally shifted to represent the same absolute distance from shock in each of the two functions. Superposition was indexed by eta-squared (η^2^), a measure of the proportion of variance accounted for by the mean of the two functions (Brown et al., 1992). When superposition is perfect, η^2^ is at its maximum value of 1.0. For each behavioral measure, η^2^ was greater under the multiplicative transform (Figure 5B, upper row) than under the additive transform (Figure 5B, lower row), and was greater than under no rescaling (Figure 5A) for freezing and respiration. The foregoing analyses were based on absolute response values for each dependent measure. When data were replotted in relative response rate (rate/overall mean rate), the results were similar, showing superior superposition with multiplicative transform for respiration (η^2^ = 0.78 vs. 0.60 for both no rescaling or additive transform), superior superposition for no rescaling for USV (η^2^ = 0.90 vs. 0.30 and 0.81 for additive and multiplicative transforms, respectively), but equivalent levels of superposition for additive and multiplicative transforms for freezing (η^2^ = 0.75 vs. 0.745, respectively, vs. 0.51 for no rescaling). Thus, the scalar property was best respected for respiration and to a lesser extent for freezing, while it was not observed for USV.

In addition, even with the superior multiplicative transform, there are discrepancies between the two CS-US interval functions, suggesting non-scalar influences on temporal response patterns. Notably, for respiration the 20-s function exhibits a transient increase at around 5–6 s after CS onset that is largely absent in the 30-s function. A similar increase in respiration was observed in the Odor group during the first half of the acquisition session (see Figure 3B, middle), but disappeared by the second half of the session (see Figure 3C middle), and then reappeared during the subsequent retention session (see Figure 4B, middle). Respiration early in the CS-US interval is consistent with exploratory sniffing of a novel odor that habituates with trial repetition and exhibits recovery between sessions. It is plausible that for the Paired group, habituation of such exploratory sniffing may compete with sensitization owing to shock delivery, but that it proceeds across sessions until only a vestige of exploratory sniffing remains on the conditioning session with the 30-s CS-US interval. The time course of the novelty response can be distinguished from the scalar temporal response pattern common to both functions.

In sum, these analyses show that the temporal pattern observed in respiration rate and freezing (although less reliably) reflects the learning of CS-US interval, and indicate that rats learned the expected time of shock delivery within 10 conditioning trials in each CS-US condition. The results obtained with the other parameter (USV, and possibly freezing) seem less clearly temporally controlled, at least during the acquisition session.

### Experiment 2: role of dopamine in the amygdala

In this experiment, we assessed the role of amygdalar dopaminergic transmission in odor fear conditioning. The D1 receptor antagonist SCH23390 was injected in the basolateral amygdala, 5 min prior to the acquisition session. The effects of this treatment on the development of temporal behavior during both acquisition and retention of learning were assessed.

#### Histological verifications

**Figure 6:**
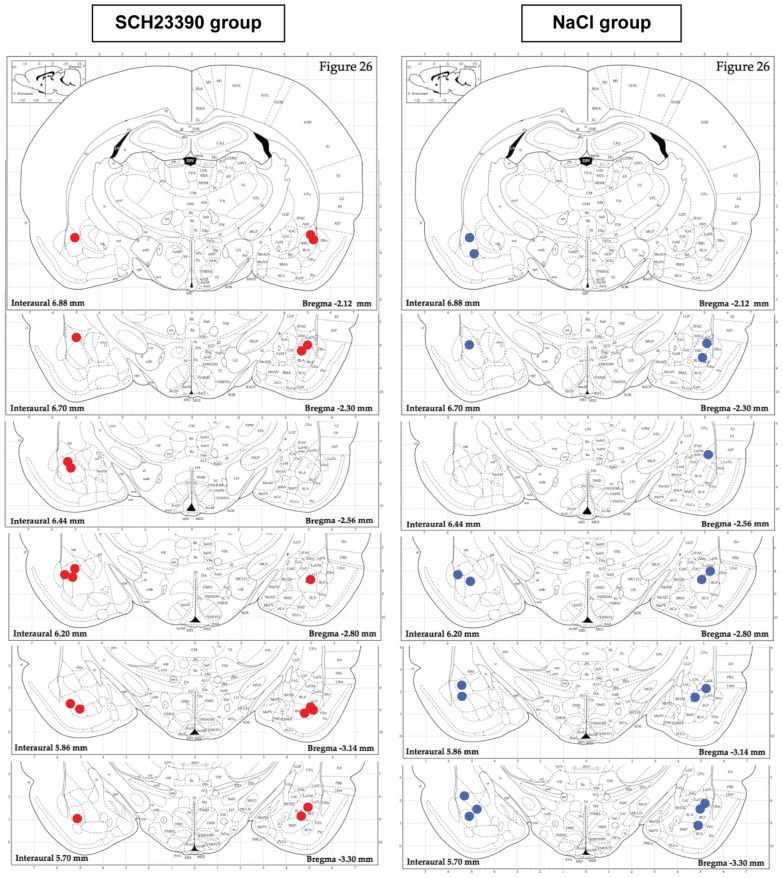
**Histological verification of injection needle placement in the NaCl and SCH 23390 groups**. Ten out of eleven animals in the SCH23390 group (red filled circles) and Ten out of thirteen animals in the NaCl group (blue filled circles) had proper injection canulae placement in the basolateral amygdala.

Figure 6 illustrates placement of injection needle tips in the basolateral amygdala in the two experimental groups. 3 animals out of 13 in the NaCl group and 1 animal out of 11 in the SCH23390 group were discarded from the experiment due to inadequate positioning of the injection needle tips.

#### Acquisition

***Global effect of conditioning.*** For each parameter the data were averaged for the 5 s preceding odor onset (Pre-CS period) and compared to the average of the 18 s between odor onset and shock delivery (CS Period) over the entire conditioning session (except the first trial). A Two Way ANOVA with one repeated measure (Period) was performed.

**Figure 7:**
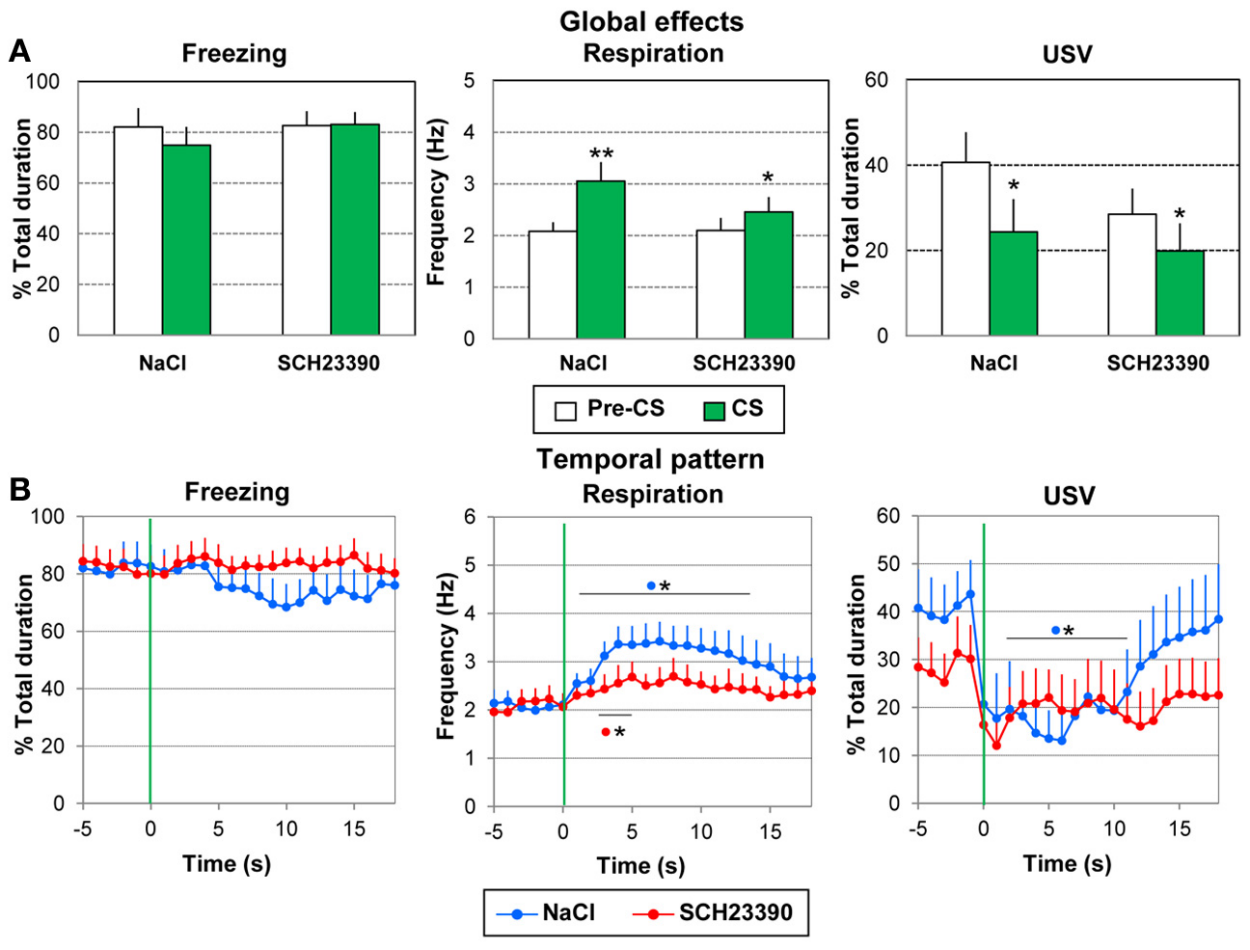
**Effect of dopamine D1 antagonist injection in the amygdala on odor-fear conditioning**. The SCH2339 group (*n* = 10) received an injection of the D1 antagonist SCH2339 just prior to the acquisition session, while the NaCl group (*n* = 10) received an equivalent amount of NaCl. In both groups, the animals received 10 odor (20 s)-shock (1 s) pairings. The data were averaged over the whole session except the first presentation. **(A)** Global effect: the data for freezing, respiration, and USV were averaged for the 5 s preceding odor onset (Pre-CS period) and compared to the average of the 18 s between odor onset and shock delivery (CS period). ^*^significant difference with Pre-CS period (^**^p < 0.005, ^*^p < 0.05). **(B)** Temporal pattern: the temporal pattern for each parameter is represented with a 1-s bin precision, from 5 s preceding odor onset (green vertical line on each graph) to 18 s after (corresponding to the CS-US interval duration). SCH23390 group: red filled circles (*n* = 10); NaCl group: blue filled circles (*n* = 10). ^*^significant difference with pre-odor baseline level (*p* < 0.05 at least). The symbol preceding the asterisk refers to the experimental group.

Concerning freezing (Figure 7A, left), the ANOVA showed no significant effect of Group, Period or Period × Group interaction. High levels of freezing were observed in the two groups, during both Pre-CS and CS periods.

For respiration (Figure 7A, middle), no significant effect of Group was observed, but a significant effect of Period [*F*_(1, 18)_ = 20.179, *p* < 0.001] and Period × Group interaction [*F*_(1, 18)_ = 4.957, *p* = 0.04]. In both groups the odor induced an increase in respiratory frequency compared to pre-CS period (*p* < 0.005 for the Saline group; *p* < 0.05 for the SCH group), with a tendency toward a larger increase for group Saline than group SCH23390 (*p* = 0.058).

Concerning USV (Figure 7A, right), 9/10 animals in the NaCl group and 7/10 animals in the SCH23390 group produced USV. The ANOVA revealed no significant effect of Group and no significant Period × Group interaction, but a significant effect of Period [*F*_(1, 14)_ = 17.677, *p* = 0.001]. In both groups the odor induced a decrease in USV emission (*p* < 0.05).

In summary, odor-shock conditioning had similar effects on the recorded parameters in both groups. It induced comparable levels of freezing and similar changes in respiration and USV in response to the learned odor.

***Temporal dynamics of the recorded parameters during the odor-shock interval.*** We then examined the temporal pattern of evolution for each parameter with 1-s bin precision, from the odor onset to shock delivery (1–18 s).

Concerning freezing behavior (Figure 7B, left), the ANOVA revealed no effect of Group, Time, or a Group × Time interaction.

Concerning respiration (Figure 7B, middle), the ANOVA revealed a significant effect of Time [*F*_(17, 306)_ = 3.39, *p* < 0.001], but no effect of Group or Group × Time interaction. Pairwise comparisons revealed that in the NaCl group, introduction of the odor induced an increase in respiratory rate which was significant from 1 to 14 s after odor onset (*p* < 0.05 at least), after which it returned to pre-odor baseline level just before shock arrival. In the SCH23390 group, the odor induced a significant increase in respiratory frequency from 3 to 5 s (*p* < 0.05).

Concerning USV (Figure 7B, right), the ANOVA revealed no effect of Group but a significant effect of Time [*F*_(17, 238)_ = 2.93, *p* < 0.001] and of Group × Time interaction [*F*_(17, 238)_ = 1.99, *p* = 0.013], In the NaCl group, introduction of the odor induced an abrupt decrease in USV production which was significant from 1 to 12 s after odor onset (*p* < 0.05). Then the USV rate returned to pre-odor level before shock delivery. In the SCH23390 group, although a slight decrease in USV was observed following odor arrival, it did not reach significance.

Thus, intra amygdala infusion of SCH23390 prior to odor fear acquisition resulted in a flattening of odor induced conditioned responses for respiration and a loss of the temporal pattern for the USV.

#### Retention test

In order to assess whether the injection of SCH23390 prior to acquisition has altered the long-term retention of learning, the animals were tested 24 h after the conditioning session in a retention test. Five presentations of the learned odor were carried out. The data from one animal in the NaCl group could not be analyzed thus resulting in an *n* = 9 in this group.

***Global effect of conditioning.*** For each parameter the data were averaged for the 5 s preceding odor onset (Pre-CS period) and compared to the average of the 40 s after odor onset (CS period, which represents the approximate duration of the odorant stimulus, as judged by the experimenter). A Two Way ANOVA with one repeated measures (Period) was performed.

**Figure 8:**
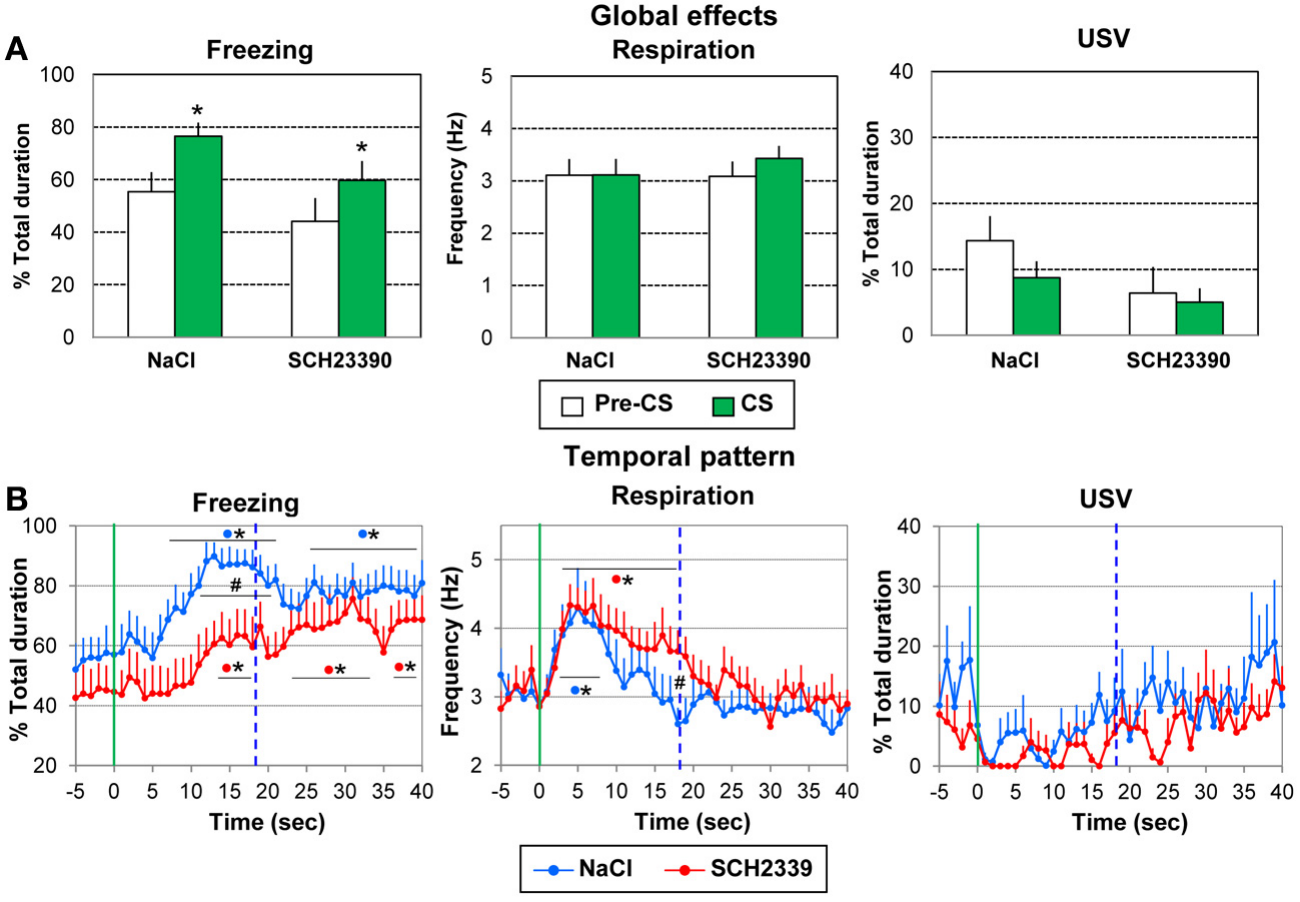
**Effect of pre-acquisition dopamine D1 antagonist injection in the amygdala on the performances during the retention session**. During the retention session, the SCH2339 group (*n* = 10) and the NaCl group (*n* = 9) received 5 presentations of the CS odor. The data were averaged over the 5 presentations. **(A)** Global effect: the data for freezing, respiration, and ultrasonic vocalization (USV) were averaged for the 5 s preceding odor onset (Pre-CS period) and compared to the average of the 40 s after odor onset (CS period). ^*^significant difference with Pre-CS period (*p* = 0.01). **(B)** Temporal pattern: the temporal pattern for each parameter is represented with a 1-s bin precision, from 5 s prior to odor onset (green vertical line on each graph) to 40 s after. The blue vertical dashed line represents the time of shock arrival in the acquisition session. SCH23390 group: red filled circles (*n* = 10); NaCl group: blue filled circles (*n* = 9). ^#^significant difference between the two experimental groups (*p* < 0.05); ^*^significant difference with pre-odor baseline level (*p* < 0.05). The symbol preceding the asterisk refers to the experimental group.

For freezing (Figure 8A, left), a significant effect of Period [*F*_(1, 17)_ = 21.13, *p* < 0.001] was observed, but no effect of Group or Period × Group interaction. In both groups introduction of the learned odor induced an increase in freezing behavior (p ≤ 0.01).

For respiration (Figure 8A, middle), the ANOVA showed no effect of Group, Period or Period × Group interaction.

Concerning USV (Figure 8A, right), 5/9 animals in the NaCl group and 5/10 animals in the SCH23390 group produced USV. No significant effect of Group, Period or Period × Group interaction was revealed by the ANOVA.

When the foregoing analyses were restricted to the 18-s CS-US interval, the conclusions regarding significant effects were unchanged.

Thus animals in both groups presented an increase in freezing in response to the learned odor suggesting that pre-acquisition injection of SCH23390 did not compromise the long-term retention of learning.

***Temporal dynamics of the recorded parameters during odor presentation.*** We then examined the temporal pattern of evolution of each parameter with a 1-s bin precision, from the odor onset to 40 s after.

For freezing behavior (Figure 8B, left), the ANOVA revealed a tendency for Group [*F*_(1, 17)_ = 3.28, *p* = 0.088], a significant effect of Time [*F*_(39, 663)_ = 12.45, *p* = 0.006] and of Group × Time interaction [*F*_(39, 663)_ = 1.68, *p* = 0.006]. In the NaCl group, introduction of the odor induced a significant increase in freezing from 8 to 21 s (*p* < 0.05) compared to baseline level, with maximum values at 15–16 s. From 26 to 40 s the freezing rate remained significantly higher than baseline levels (*p* < 0.05). In the SCH23390 group, the odor induced a slowly developing increase in freezing which reached significant levels from 13 to 17 s, and later from 23 to 34 s (*p* < 0.05). A maximum in freezing was observed at 32 s. Between groups comparisons showed that the level of freezing was higher in the NaCl group than in the SCH23390 group from 13 to 17 s (*p* < 0.05).

Concerning respiration (Figure 8B, middle), the ANOVA revealed no effect of Group but a significant effect of Time [*F*_(39, 663)_ = 10.80, *p* < 0.001]. In both groups, arrival of the odor induced a significant increase in respiratory rate (*p* < 0.05, from 2 to 8 s in the NaCl group, from 3 to 17 s in the SCH23390 group), after which the respiratory frequency decreased to baseline level showing a minimum value for 18 s in the NaCl group and 30 s in the SCH23390 group.

Finally, when considering USV (Figure 8B, right), 5/9 animals in the NaCl group and 5/10 animals in the SCH23390 group produced USV. The ANOVA revealed no effect of Group, Time, or Group × Time interaction.

When the foregoing analyses were restricted to the 18-s CS-US interval, the conclusions regarding significant effects were globally unchanged.

Thus, while the global increase in freezing in response to the learned odor was similar in both groups, the temporal pattern of freezing was different in SCH23390 animals compared to NaCl animals. The same tendency was observed for respiration.

## Discussion

The present study was aimed at investigating whether and when time durations are encoded in odor fear conditioning, and at assessing the role of DA in the amygdala in the CS-US interval time processing. The key findings are that (1) using complementary behavioral and physiological indices, interval timing can be inferred from the animal's behavior during the acquisition session after a few training trials and (2) D1 receptors DAergic transmission in the amygdala is involved in timing the CS-US interval. More specifically, we carried out a fine-grain analysis of the time-course of the animal's fear response using the monitoring of three complementary indices: freezing behavior, respiratory activity and USV production. The data show that after a few odor-shock pairings, a temporal pattern of responding related to CS-US interval duration could be detected during the acquisition session, characterized by a decrease in freezing, a decrease in respiratory rate and an increase in USVs emission just prior to shock arrival. We demonstrated for one of the measures (respiration) that this temporal pattern was linked to interval timing since changing the CS-US interval duration resulted in a proportional shift of the temporal response toward the new duration value. When tested for their retention of the learning 24 h later, the animals showed timed anticipatory responses characterized by an increase in freezing and a decrease in respiratory rate around the expected time of arrival of the shock, suggesting they have stored the duration in their long-term memory. Finally we showed that blocking dopamine D1 receptors in the amygdala resulted in an alteration of timing behavior during both the acquisition and retention sessions.

### Changing interval duration results in a shift of the temporal pattern toward the new duration

It could be argued that the temporal pattern observed during acquisition is due to other factors than timing behavior. In order to discard this possibility, we examined the effect of changing the interval duration on the previously observed temporal pattern. We showed that after five trials with the new duration, the decrease in respiratory rate and in freezing were shifted toward the new time of arrival for the shock. Interestingly the new temporal pattern seems to respect scalar property. Indeed, one of the fundamental properties of interval timing is that variability in the timing behavior of an animal grows proportionally with the duration of the timed stimulus. This has been termed the scalar property (Gibbon, 1997). In our study, the width of the temporal pattern observed for a 30 s CS-US interval is larger than for the 20 s interval, and functions superimposed when the time axis was normalized. For these two reasons (shift toward new duration and scalar property) we assume that this behavioral temporal pattern is related to timing behavior.

The scalar property was not observed for all three measures. For USV, superposition between functions for the two CS-US intervals was best when the time axis was not shifted, implying a temporal pattern that was triggered by the onset of the odor but not controlled by expectancy of shock. However, this measure was compromised by small number of subjects contributing vocalization data that may be insufficiently stable to show the scalar effect.

The measurement of temporal patterns was further challenged by influences on performance arising from non-temporal sources. For instance, habituation to novelty appeared to contribute to the pattern of performance on the respiration measure, and the temporal pattern of freezing during the CS changed from a decreasing trend during acquisition to an increasing trend during retention, likely attributable to extinction of freezing to the context between the two sessions. It is known that the temporal form of the conditioned response may change with amount of training (e.g., Ellison, 1964). The present data suggest that this change may involve complex interactions among several processes, but they also demonstrate that the scalar property may be detected even in the earliest stages of development of the response.

### Interval duration is learned early during training and stored in long-term memory

Previous studies of Pavlovian conditioning with appetitive protocols in birds have demonstrated temporal superposition of acquired behavioral repertoires after 20–125 conditioning sessions (Brown et al., 1997; Ohyama and Mauk, 2001). The present study extends that finding to the aversive case in rats, within a single conditioning session. Our data show that from the second half of the session, a timed anticipatory response can be detected during the CS-US interval. This observation confirms and extends previous data showing that encoding of interval durations in fear conditioning seems to occur early during conditioning (Davis et al., 1989; Drew et al., 2005). Davis et al. (1989) trained rats with 30 light-shock pairings using a wide range of CS-US intervals and assessed conditioned fear using the fear-potentiated startle effect. The data showed that for a variety of test intervals, the test interval that induced maximal potentiated startle was generally close to, or identical with, the CS-US interval used in training. The authors reported that temporal specificity developed over a very small number of training trials. In the Davis et al. (1989) study, the temporal pattern of fear response was assessed during the retention test. In the present study, recording the three parameters in parallel allowed us to track the development of temporal response patterns during the acquisition session yielding information regarding the developmental dynamics of this temporal pattern. We observed that by the second half of the session a temporal pattern was clearly visible (mainly for respiration) which respected scalar property rules. When tested 24 h later for their retention of the learning, the Paired animals exhibited a temporal pattern of fear response parameters around the expected time of shock arrival suggesting they have stored the duration in long-term memory.

### Freezing, respiration and USV provide different timing-related information during acquisition vs. retention

Our results show that respiration, USV and freezing bring complementary information and are differentially affected between acquisition and retention. More specifically during acquisition, respiration proved to be a good index to assess interval timing. Indeed, respiration rate decreased a few seconds before shock arrival. Freezing seemed a less reliable parameter although it showed some temporal pattern mainly when the number of pairings was increased (for the change in interval duration). In contrast, a decrease in USV may have been mainly driven by the CS onset and may not be controlled by the temporal expectation of the US. During the retention test carried out 24 h after training, timing can be inferred from freezing and respiration rate which exhibit, respectively, an increase and decrease in level around the expected time for the shock. Thus, from the three recorded parameters, respiration seems the most reliable index of timing behavior, since it is temporally modulated both during acquisition and retention.

Respiration is a highly phasic signal: in the first seconds after odor onset, respiration rate is changed due to the sampling of the stimulus. Then as the animal recognizes the stimulus and its learned significance, respiration rate is affected by the emotional value of the stimulus. Indeed previous studies have shown that when a neutral odor is paired with a positive reward in a classical conditioning paradigm, high-frequency sniffing develops in anticipation of reward delivery (Freeman et al., 1983; Monod et al., 1989; Kepecs et al., 2007; Wesson et al., 2008). Interestingly, Kepecs et al. (2007) showed that reward-anticipatory sniffing occurred in a higher-frequency range (9–12 Hz) compared with odor sampling sniffing (6–9 Hz). In our case, while odor sampling sniffing is around 8–9 Hz as assessed during the first trial of odor presentation, the anticipation of a negative reward (shock delivery) resulted in a lowering of respiratory frequency (3–5 Hz) which reached a minimum value just prior to shock arrival. To what extent the observed temporal pattern for respiration is specific to odor stimuli or could also be obtained using other sensory stimuli is an issue that would deserve further investigation.

During acquisition, the temporal pattern of USV appeared to be a mirror image of that for respiration. When the conditioned odor cue was delivered, USVs ceased and were produced again just before shock arrival. Previous studies have shown that USVs are emitted during expiration and strongly constrain the respiration frequency (Frysztak and Neafsey, 1991; Hegoburu et al., 2011), consistent with the pattern we observed during acquisition. However, unlike respiration, USV did not yield evidence of scalar timing, and during the retention test the CS produced a decrease in both measures, suggesting that respiration and USV may be partially dissociable. USV levels remained higher during the ITI than during the CS throughout acquisition and retention tests. It has been suggested that context cues in the ITI, remote from an aversive US may signal anxiety, whereas the CS contiguous with the US may signal acute fear (Jelen et al., 2003), and that USV may specifically reflect the anxiety state. While freezing rate is often similar in fear and anxiety states, sustained 22-kHz USV have been shown to occur preferentially between trials, whereas acute fear induced by the CS as an imminent danger signal resulted in immediate inhibition of USV (Frysztak and Neafsey, 1991; Jelen et al., 2003). The present results are consistent with prior findings suggesting suppression by the odor CS of context-supported USV.

The dynamics of the freezing response differed from those of both respiration and USV. During acquisition, freezing was close to a ceiling level after a few odor-shock pairings presumably reflecting context conditioning (Fanselow, 1980; Wood and Anagnostaras, 2011), and thus could not exhibit further increases in level during the CS. During the retention test, the level of freezing during the ITI was lower than during acquisition, revealing a temporal pattern of increased freezing during the CS, with a maximum around the expected time of shock arrival. It is possible that testing the retention (instead of the acquisition as presented here) of an altered CS-US interval would provide evidence of scalar timing with the freezing measure.

Thus, the three recorded parameters provide complementary information related to interval timing depending on the learning phase considered (acquisition or retention), and can be used accordingly in order to increase the likelihood of detecting temporal pattern in animal's fear response.

### D1 receptors dopamine transmission in the amygdala is involved in the modulation of temporal encoding/memory

In Experiment 2, we investigated the role of basolateral amygdalar dopaminergic transmission in CS-US interval timing in odor fear conditioning. Research on the neural basis of timing has suggested that DA plays an important role. For example, Schultz and colleagues have found that the firing of midbrain dopamine neurons represents temporal expectations about reward (Hollerman and Schultz, 1998). Moreover, the administration of systemic D2 receptors DAergic agonists or antagonists has been shown to alter the animals' expectations about the time of reward availability (Maricq et al., 1981; Frederick and Allen, 1996; Meck, 1996; Buhusi and Meck, 2002; Drew et al., 2003). In general, DAergic antagonists cause underestimation of time, and agonists cause overestimation of time, although the sensitivity to different agonists and antagonists may differ depending on the temporal task used (Body et al., 2013). In all these studies, however, general administration (i.p. or s.c.) of the drugs does not provide information about the site of action, which may differ for each drug and task combination condition. Here we show that intra-amygdala infusion of the D1 DAergic receptors antagonist SCH23390 alters the temporal pattern during acquisition and causes the peak in responding to occur later in the trial during the retention test. This latter observation is in accordance with the effects previously described using systemic injections of DAergic antagonists although these have been obtained using D2 DAergic receptors antagonists.

It might be argued that the effect of SCH23390 on temporal pattern should rather be ascribed to a deficit in associative learning. Indeed dopamine in the amygdala plays an important role in fear and anxiety (for a recent review, see de la Mora et al., 2010). For instance, it has been reported that stress induced an increase in dopamine release in the amygdala (Abercrombie et al., 1989; Young and Rees, 1998; Inglis and Moghaddam, 1999; Yokoyama et al., 2005). In addition, pretraining infusion of SCH23390 within the amygdala before tone fear conditioning was shown to decrease learned fear at testing (Guarraci et al., 1999). In line with this set of data, Rosenkranz and Grace (2002) using an odor fear conditioning protocol on anesthetized rats, found that repeated pairings of an odor with a foot-shock resulted in neuronal plasticity in the lateral nucleus of the amygdala. This plasticity was blocked by the local infusion of the dopamine antagonist haloperidol, suggesting that dopaminergic transmission in the amygdala is also involved in odor fear conditioning. However, in the present study, although pre-training infusion of SCH23390 in the amygdala induced a flattening of the odor induced conditioned responses when the animals were under the drug, it did not alter the global level of learned fear as assessed during the retention session, when the animals were off the drug. This discrepancy with the results reported by Guarraci et al. (1999) might be explained by the higher number of CS-US pairings in our study (10 vs. 3), which might have rendered the learning more resistant to the dopaminergic antagonist. Therefore, it can be assumed from the present data that D1 receptor dopaminergic transmission within the amygdala is involved in CS-US interval processing in fear conditioning.

Our study confirms previous data suggesting that the amygdala could be involved in interval timing (for a review, see Diaz-Mataix et al., 2013b). In a recent experiment carried out on monkeys, Bermudez et al. (2012) using visual stimuli predicting different momentary probabilities of reward occurrence that resulted in specific temporal reward structures, reported that neurons in the amygdala were sensitive to the expected time of reward. These data suggest an active involvement of amygdala neurons in timing processes that are crucial for reward function. In another recent study, Diaz-Mataix et al. (2013b) trained rats in an auditory fear conditioning protocol in which a tone CS was associated with a foot-shock US delivered 30 s after the tone onset. The animals received ten CS-US pairings followed 24 h later by a reactivation trial consisting of either a single CS-US pairing identical to the training condition, or a single pairing in which CS-US time interval was reduced from 30 to 10 s. In both groups, an intra-amygdala infusion of a protein synthesis inhibitor was carried out immediately after reactivation. This treatment induced a memory deficit only in the animals that experienced the shock at the new time. Therefore, changing the interval duration between the CS and the US during reactivation was sufficient to trigger protein-dependent synaptic plasticity and reconsolidation of the memory in the amygdala. These data mean that the animals had encoded the CS-US interval duration in the original learning and that a single presentation of the shock at a new time was enough to trigger an updating of the memory involving amygdala-dependent synaptic plasticity (Bailey and Balsam, 2013). The present study brings further arguments to the involvement of amygdala in time processing and adds the new information that this might be supported by D1 dopaminergic receptors signaling.

## Conclusion

The literature of timing suggests that timing intervals in the seconds-to-minutes range involves the activation of a large network of brain areas that form part of the thalamo-cortico-striatal circuits including the basal ganglia, the prefrontal cortex and the posterior parietal cortex (for a review see Buhusi and Meck, 2005). The present study suggests that the amygdala might also take part in this network. In addition, behavioral and neurophysiological data have highlighted the functional contribution of sensory-specific cortices and support the existence of modality-specific timing mechanisms (Bueti, 2011). In accordance with this hypothesis, in a previous study investigating the dynamics of Glutamate and GABA in the olfactory cortex during odor fear acquisition, we described a temporal pattern of both amino acids release occurring in the olfactory cortex after the last odor–shock pairing, at the predicted times of anticipated trials (Hegoburu et al., 2009). This led us to propose that the olfactory cortex might store certain aspects of fear conditioning related to the timing of the associations. Previous studies have shown that the piriform cortex is involved in odor fear conditioning (Sevelinges et al., 2004, 2008, 2011; Moriceau et al., 2006; Jones et al., 2007; Barnes et al., 2011; Chen et al., 2011) and contains a high density of dopaminergic axons (Datiche and Cattarelli, 1996) and D1 dopaminergic receptors (Maltais et al., 2000; Zenko et al., 2011; Garske et al., 2013). Therefore, the piriform cortex could represent another good candidate for time processing in odor fear conditioning. Whether and how the amygdala and olfactory cortex work in concert to process time during associative odor fear learning is an interesting issue that would deserve further investigation.

### Conflict of interest statement

The authors declare that the research was conducted in the absence of any commercial or financial relationships that could be construed as a potential conflict of interest.
